# Effect of internal limiting membrane peeling on normal retinal function evaluated by microperimetry-3

**DOI:** 10.1186/s12886-020-01383-3

**Published:** 2020-04-09

**Authors:** Yue Qi, Zengyi Wang, Shi-Ming Li, Qisheng You, Xida Liang, Yanping Yu, Wu Liu

**Affiliations:** 1 Department of Ophthalmology, Beijing Tongren Eye Center, Beijing Ophthalmology and Visual Sciences Key Lab, Beijing Tongren Hospital, Capital Medical University, 1 Dongjiaomminxiang Street, Dongcheng District, Beijing, 100730 China; 2 Beijing Institute of Ophthalmology, Beijing Tongren Eye Center, Beijing Ophthalmology and Visual Science Key Lab; Beijing Tongren Hospital, Capital Medical University, Beijing, China

**Keywords:** Internal limiting membrane peeling, Microperimetry-3, Retinal sensitivity, Macular hole, Vitrectomy

## Abstract

**Background:**

To evaluate the effect of internal limiting membrane (ILM) peeling surrounding macular holes (MH) for the function of retina by microperimetry-3(MP-3).

**Methods:**

This is a prospective, cohort study which included patients with MHs who were treated by 23-gauge 3-port pars plana vitrectomy and ILM peeling with air tamponade. Color fundus photography, retinal optical coherence tomography and MP-3 were performed 1 week before, 1 and 4 months after the operation. In MP-3 examination, a customized follow-up pattern with 45 spots in the central 8° visual field was used. The spots corresponding to the retina surrounding macular holes were selected for comparison of pre- and post-operative function.

**Results:**

We incuded 44 eyes of 44 patients with best corrected visual acuity (BCVA) of 1.06 ± 0.40 (logMAR). All eyes achieved an anatomical success at 4 months. BCVA significantly improved at 1 month (0.53 ± 0.30, *P* < 0.01) and 4 months (0.31 ± 0.24, *P* < 0.01), respectively. Mean retinal sensitivity (MRS, dB) of the retina surrounding macular hole was 23.46 ± 3.01 dB at baseline, and significantly increased at 1 month (26.25 ± 2.31 dB, u = − 4.88, *P* < 0.01) and 4 months (27.14 ± 2.45 dB, t = − 6.29, *P* < 0.01). Patients with increased MRS are significantly younger than those with deceased MRS (59.72 ± 3.22 years vs. 65.60 ± 8.19 years, *P* < 0.01). After ILM peeling, the increasing extent of MRS was significantly higher in inferior and nasal retina than in superior and temporal retina at 1 and 4 months (*P* < 0.05).

**Conclusion:**

ILM peeling in normal retina will not decrease the retinal function in a short-term after surgery.

## Background

Internal limiting membrane (ILM) peeling has been considered as an important procedure to increase the anatomic success rate in surgeries for macular diseases such as macular hole or epi-retinal membrane. IML peeling may release the tangential traction to the retina of macular area, and activate Müller cells, stimulating the secretion of collagen, basement membrane components, inflammatory factors which may stimulate glial cell-mediated closure of macular holes (MH) [1]. However, the use of ILM peeling in macular surgery is still controversial.

Controversy focused on the potential side effects of ILM-peeling. Major side effects of ILM peeling have been reported as potential mechanical or functional damage to retina [2–10]. Previous studies about retinal functional changes that caused by ILM peeling are conflicting: some studies reported no changes after peeling [11, 12], whereas others showed decrease of retinal sensitivity [13, 14].

In previous studies about the effect of ILM peeling to MRS, the selected regions generally contained all the ILM peeling areas which also including MH area. The results may be easily affected by the changes of macular lesion itself and could not provide a strong evidence of influence of ILM peeling to retinal function. Whether ILM peeling may damage retinal function? The purpose of this study was therefore to investigate, in eyes with MH, the influence of ILM peeling to normal retinal function (outside the area of macular hole), using the newest type of microperimetry (MP3) combined with spectral domain optical coherence tomography (SD-OCT).

## Methods

In this prospective cohort study, 44 eyes of 44 patients with idiopathic MH were evaluated in Beijing Tongren Eye Center, Beijing Ophthalmology and Visual Science Key Lab; Beijing Tongren Hospital from November 2016 to April 2017. The Medical Ethics Committee of the Beijing Tongren Hospital approved the study protocol, and all participants gave their written informed consent. Color fundus photography, retinal optical coherence tomography (OCT) (Carl Zeiss, Dublin, CA, USA) and microperimetry-3 (NIDEK, Gamagori, Japan) were performed for each patient 1 week before, 1 and 4 months after operation. MH was ensured by OCT. We defined the diameter of a MH as the minimum diameter.

The inclusion criteria for patients included a diagnose of idiopathic MH conformed by OCT, a requirement of operation for treatment, and opacities of lens under NO3C2P1 grade assessed by Lens Opacities Classification System III (LOCSIII). Meanwhile, patients with glaucoma, myopia<− 3.0 diopters (D), severe cataract, or other ocular diseases that could interfere with the measurements were excluded.

A standard 23-gauge 3-port pars plana vitrectomy was performed by the same experienced surgeon (W.L.). Phacoemulsification and IOL implantation were performed if necessary. A subtotal vitrectomy was performed followed by IML peeling without staining. The posterior hyaloid was elevated and trimmed in all patients. The ILM was peeled off with forceps in an area of about 2 disc diameter around the MH. A fluid–gas exchange was carried out, and the vitreous cavity was filled with air. All operations were performed without any serious postoperative complications. Patients were asked to stay in a prone position for 5–7 days after surgery. At 1 and 4 months after surgery, patients returned for a follow-up visit with examination of color fundus photography, optical coherence tomography (OCT) (confirming the closure of the MH) and microperimetry-3.

Retinal function of patient was evaluated by microperimetry (MP) which was a subjective, quantitative, non-invasive diagnostic exam aimed at assessing retinal functionality and to put it in strict correlation with retinal morphology. Microperimetry-3 (MP-3), as the newest generation of microperimetry, has a wider range of stimulus intensity from 0 to 34 dB. MP-3 can measure perimetric threshold values even for normal eyes. A maximum stimulus luminance of 10,000 asb allows evaluation of low-sensitivity. The MP-3 device features faster tracking, increased automation and a broader dynamic range compared with the MP-1 [9]. Another important feature of this microperimeter is that target light is projected onto the retina rather than a screen. The position of the retina is therefore tracked so that target presentations can be automatically aligned, and the exact same location is stimulated at each target presentation. In this manner, we would expect to observe highly reproducible measurements of retinal sensitivity [10].

**Fig. 1:**
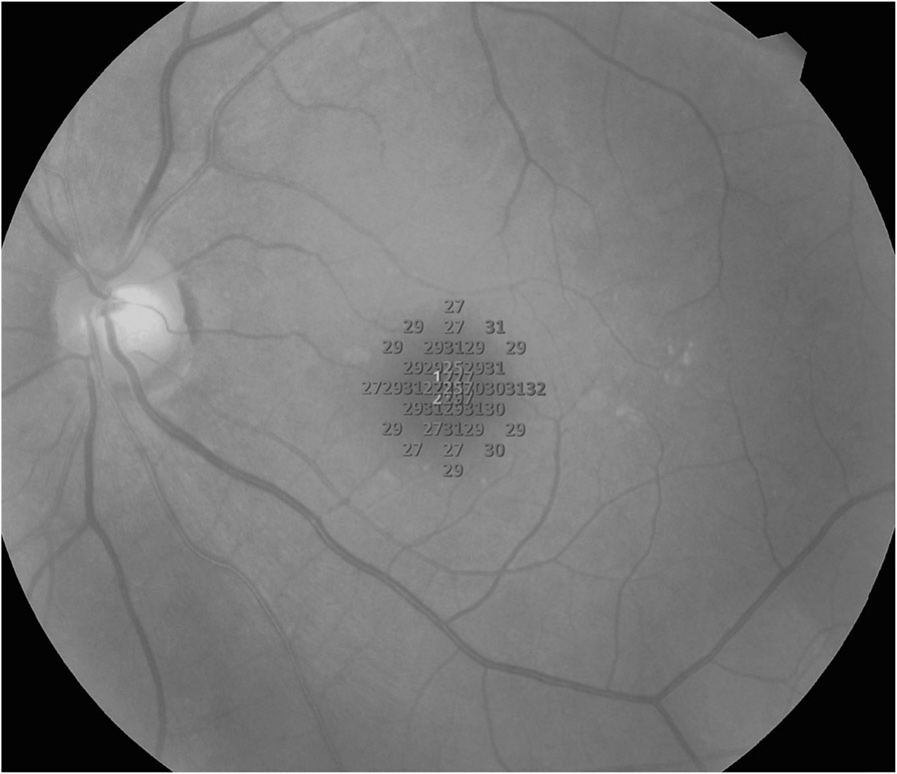
A customized pattern was used in 8° of the visual field, with 45 spots

The microperimetry examination was performed in a dark room. All patients underwent a dark adaptation for at least half an hour until the pupil size reached 4 mm or larger. The infrared fundus image was registered, and the central fixation point was aligned to the center of MH in pre-operative examination. The follow-up pattern was used to make sure the pre- and post-operative examinations and comparisons were point to point perfectly matched. A customized pattern with 45 spots in central 8° visual field was used. The 45 test points in the MP-3 are shown in Fig. 1.

**Fig. 2:**
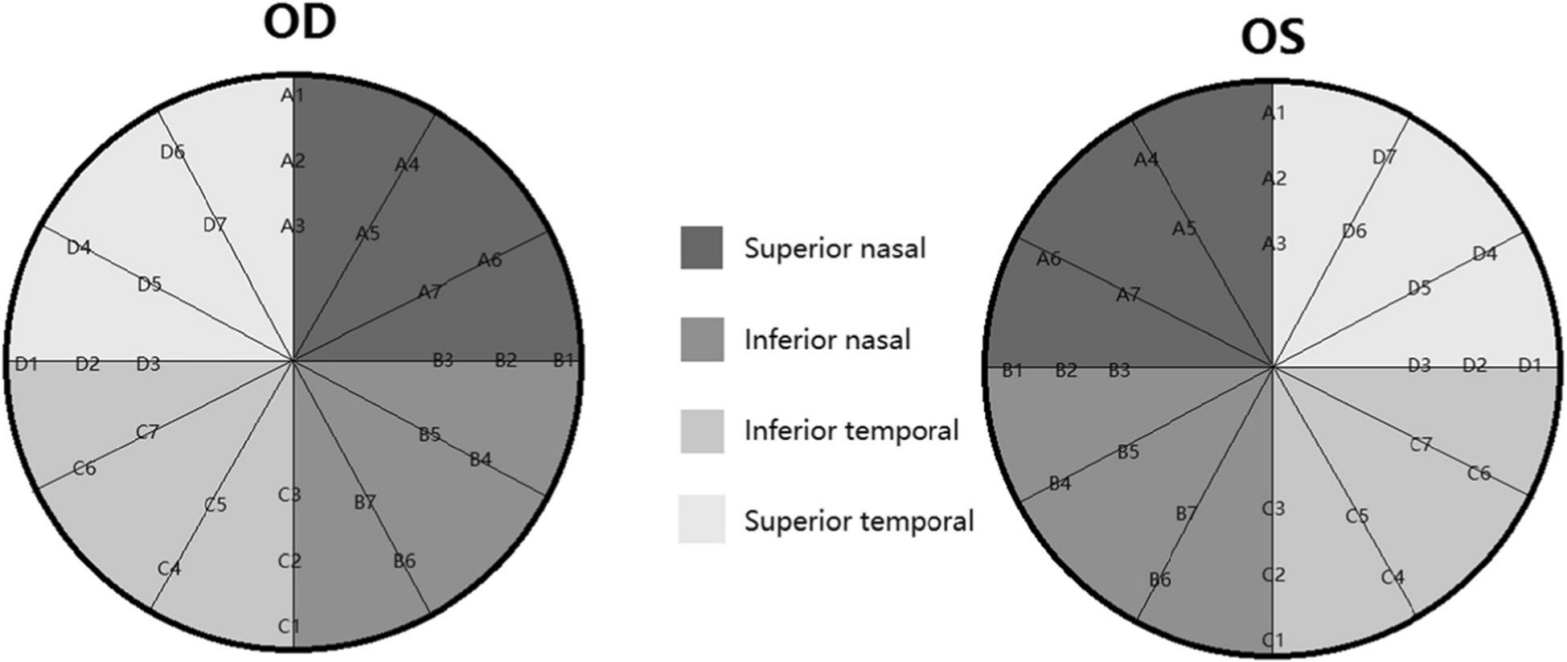
The area was divided into four regions, superior nasal (point A1–8), inferior nasal (point B1–8), inferior temporal (point C1–8), and superior temporal (point D1–8). When calculating, the points located on X-axis or Y-axis were excluded. For example, when comparing the retinal sensitivity between superior and inferior retina, the points located on X-axis (point B1,2,3 and D1,2,3) were excluded. In the same way, when comparing the retinal sensitivity between nasal and temporal retina, the points located on Y-axis (point A1,2,3 and C1,2,3) were excluded

The fixation target was a 1° diameter red circle, and the background luminance was set at 31.4 asb, giving suitable evaluation of macular sensitivity and enabling detection of small visual field defects in the macular area. Only reliable VFs were used in analyses, which were defined as fixation loss (FL) rate < 20% and a false-positive (FP) rate < 15%. We used a Goldman size III stimulus with duration of 200 ms. Using the obtained retinal sensitivities, the mean sensitivity at the fovea, within two degrees, four degrees, six degrees and eight degrees were calculated. Four regions, superior nasal, inferior nasal, inferior temporal, superior temporal, were divided and shown in Fig. 2.

**Fig. 3:**
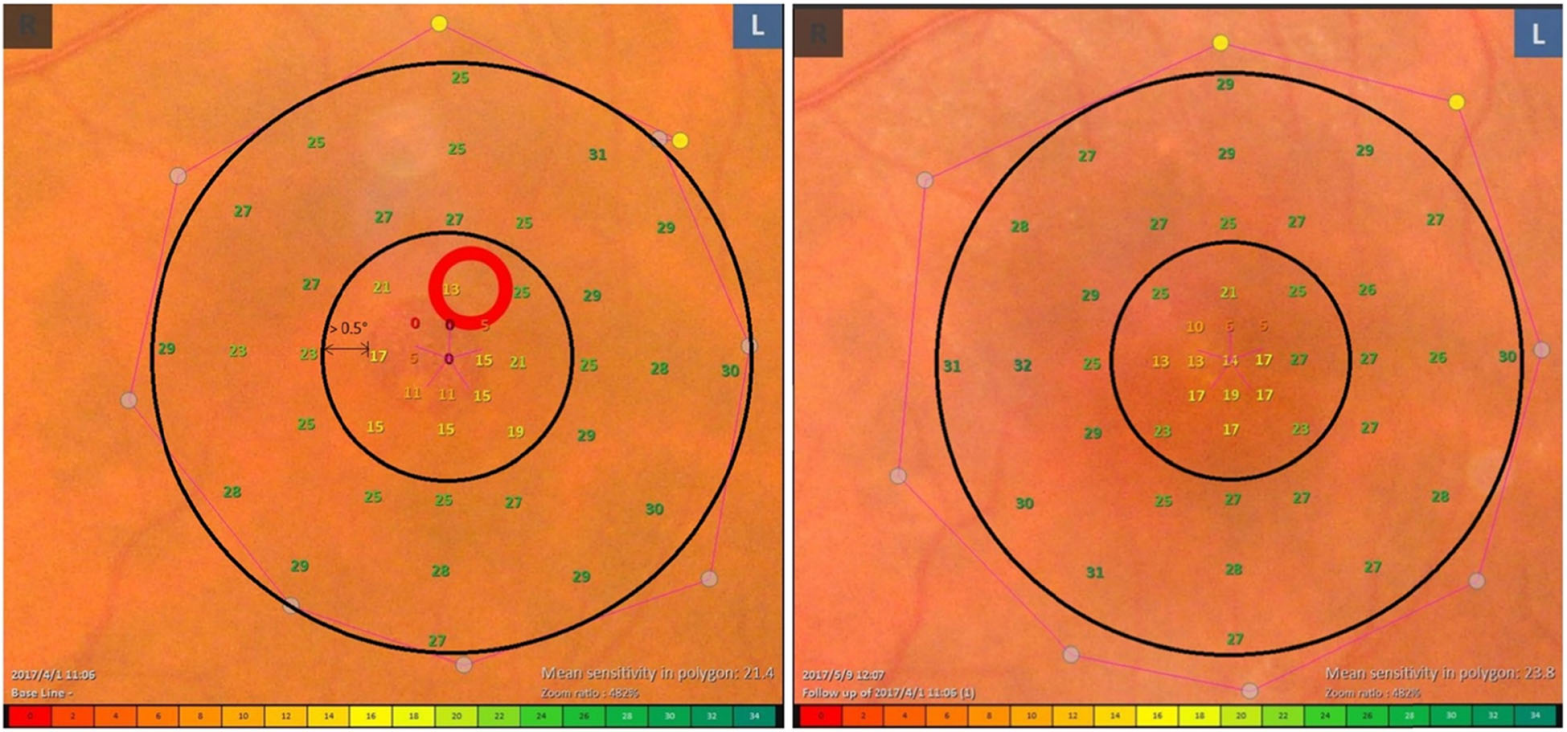
Left eye of a Chinese patient (60–70 years old) with a macular hole (MH) treated by pars plana vitrectomy with ILM peeling. (left) Preoperative; (right) Postoperative; The examiner was asked to fix the fixation target to the central of MHs as much as possible. Follow-up pattern was used to ensure the selected dots located on the same position whether in preoperative or in postoperative examination. When calculating, we only choose 28 points in the outer ring zone (between the two black circles) instead of all 45 points, which located in the normal retina, and the area covered by MH was excluded

**Fig. 4:**
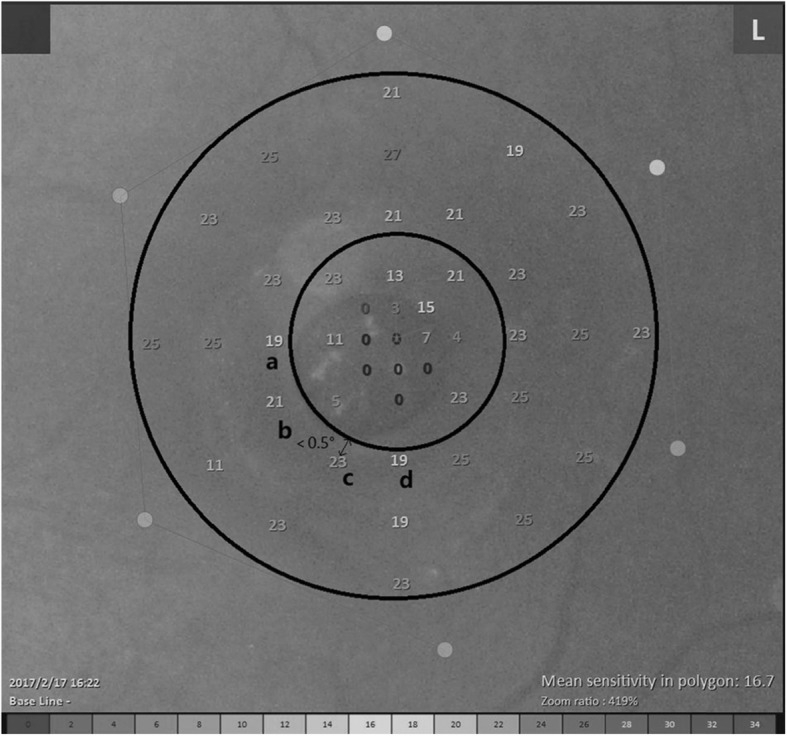
Left eye of a Chinese patient (60–70 years old) with a huge macular hole (MH). Points a, b, c, d was excluded for the distance from these points to the margin of MH was less than 0.5°. When calculated for this patient, we only chose 24 points in the outer ring zone

When calculating, the points located on X-axis or Y-axis were excluded. We choose 28 points within the outer ring zone covering the normal retina, not including the 17 points for MHs area (Fig. 3). These 28 points occupied 60% of the whole 45 points, and covered more than 75% area of the 8° retina. These points located from 4° to 8°. The diameter of 8° visual field was 2500 μm (about 1.6PD). During the operation, the ILM we peeled off covered an area of at least 2 PD, which meant the 8° area was completely contained in the ILM peeling area. The point with a distance of less than 0.5° from the margin of MH was also excluded (Fig. 4).

We used follow-up pattern to ensure the selected dots located on the same position in every examination. All tests were conducted by one experienced microperimetry examiner. On the basis of the microperimetry findings, we evaluated mean retinal sensitivity (primary outcome) of all the selected points.

### Statistical analysis

Statistical analysis was performed using a commercially available statistical software package (SPSS for Windows, version 25.0, IBM-SPSS, Chicago, IL, USA). BCVA measurements were converted to the logarithm of the minimum angle of resolution (LogMAR). The parameters are presented as mean ± standard deviations. Pre- and post-operative visual acuities and retinal sensitivity were compared using paired Student’s *t* test (in the normal distributed samples) or *Mann-Whitney U test* (in the non-normal distributed samples)*.* A *P*-value < 0.05 was considered statistically significant.

## Results

MHs were closed in 42 eyes within a single operation. Two eyes underwent vitrectomy again with air tamponade at 1 week due to unclosed MHs after the first surgery. At 1 month, all eyes achieved an anatomical success of closed MHs without postoperative complications. The characteristics of the patients and MHs are presented in Table 1.

**Table 1:** Characteristics of the patients and MHs

	Mean (Range)
Age (years)	64.25 ± 5.48
Preoperative BCVA (logMAR)	1.06 ± 0.40 (0.01–0.60)
Diameter of MHs (μm)	535.72 ± 164.17 (230–864)
	n(%)
Staging	
1	0
2	9
3	24
4	11
Sex	
1	10 (23)
2	34 (77)
Laterality	
Right eye	21 (48)
Left eye	23 (52)
Preoperative lens status	
Pseudophakia	0 (0)
Clear lens	9 (20)
Cataractous	35 (80)

*Abbreviations*: *BCVA* best-corrected visual acuity, *MH* macular hole

Mean BCVA (logMAR) was 1.06 ± 0.40 before surgery, and significantly increased at 1 month (0.53 ± 0.30, t = 7.03, *P* < 0.01) and 4 months (0.31 ± 0.24, t = 10.66, *P* < 0.01), with that of 4 months increased more than that of 1 month (t = 3.80, *P* < 0.01). The number of selected points ranged from 22 to 28 (mean: 26.3 ± 1.8).

Mean retinal sensitivity (MRS) of the selected area was 23.46 ± 3.01 dB before surgery, and significantly increased at 1 month (26.25 ± 2.31 dB, u = − 4.88, *P* < 0.01) and 4 months (27.14 ± 2.45 dB, t = − 6.29, *P* < 0.01) after ILM peeling. There was no difference in MRS between 1 and 4 months after surgery (t = − 1.75, *P* = 0.08).

During the 4 months, there was no significant change in MRS for the fellow eyes without MHs (27.97 ± 1.66 dB vs. 28.33 ± 1.40 dB, t = 1.26, *P* = 0.45). Compared with the fellow eyes without MHs, the eyes underwent surgery had significantly lower MRS in selected 28 points before surgery (23.57 ± 2.62 dB vs. 27.97 ± 1.66 dB, t = 18.02, *P* < 0.01), and had an improvement in MRS achieving to a similar level at 4 months postoperatively (27.55 ± 1.71 dB vs. 28.33 ± 1.40 dB, t = 4.25, *P* = 0.10).

Post-operative MRS in the selected normal retinal area increased in 37 patients but deceased in 7 patients. Patients with increased MRS were significantly younger than patients with deceased MRS (59.72 ± 3.22 years vs. 65.60 ± 8.19 years, t = − 4.98, *P* < 0.01). Phacoemulsification and IOL implantation was performed in 35 eyes. The increasing extent of MRS was not significantly different between patients with phacoemulsification and those without at 1 month (2.77 ± 3.29 vs. 2.81 ± 1.86, t = − 1.50, *P* = 0.97) and at 4 months (3.46 ± 3.01 vs. 3.88 ± 1.02, t = − 0.36, *P* = 0.25).

Before surgery, there was no significantly difference in MRS between superior and inferior retina (23.89 ± 2.34 dB vs. 23.01 ± 4.01 dB, t = 1.26, *p* = 0.10), or between nasal and temporal retina (23.15 ± 7.12 dB vs. 23.74 ± 4.13 dB, t = − 0.48, *P* = 0.19). After ILM peeling, the increasing extent of MRS was significantly higher in inferior retina than in superior retina at 1 month (*P* = 0.03) and 4 months (*P* = 0.01). Also, the increasing extent of MRS was significantly higher in nasal retina than in temporal retina at both 1 and 4 months (*P* < 0.001and *P* = 0.03).

We did statistic analyze (OR) for factors might be associated with the treatment results and found longevity (*P* = 0.348), BCVA (*P* = 0.209), macular hole diameter (*P* = 0.649) and course of disease (*P* = 0.174) all showed no correlation with retinal sensitivity after surgery.

## Discussion

ILM peeling has been considered as a useful technique in surgeries for vitreomacular interface diseases. It has been reported that the macular hole closure rate was 90–100% when treated with vitrectomy and ILM peeling, while it was only 60–90% without ILM peeling [15–18]. However, potential damages to retinal function caused by ILM peeling was considered as a side effect of this technique.

The findings of previous studies about influence of ILM peeling on retinal function were controversial. Some studies evaluated a dissociated optic nerve fiber layer (DONFL) in ILM-peeling area and found the retinal function in this area did not changed after surgery. Yasuki et al. [7] compared the retinal sensitivity of DONFL area and non-DONFL area in twenty ILM-peeled eyes with MH more than 4 months after the vitrectomy by scanning laser ophthalmoscopy (SLO) microperimetry. Yoshinori et al. [11] performed static microperimetry-1 in 31 eyes with MH and receiving vitrectomy to explore the possible relationship between the DONFL appearance and retinal function. Hiroki et al. [12] investigated the effects of DONFL on retinal sensitivity in 17 eyes with an idiopathic macular hole that underwent vitrectomy and internal limiting membrane (ILM) peeling. They all found DONFL associated with ILM peeling does not alter retinal function in the area of the DONFL. While all these studies focused on the changes of retinal structure (DONFL) followed by ILM peeling, the results can only prove that the function of DONFL area, instead of ILM peeling area, had not been injured. In the current study, the DONFL was observed in 3 patients, which was only 7.1% of all cases. Therefore, the existence of DONFL can not be the main reason to interfere the retinal function in the current case series. In this study, we mainly discussed the retinal function in ILM-peeling area instead of the DONFL area. The detection method was also different with previous studies. So, the changes of retinal function in DONFL area can not be evaluated in this article.

Other studies supported that retinal function decreased after ILM-peeling. Terasaki et al. [19] analyzed recordings of focal macular electroretinograms (FMERGs), observing retinal physiology in the macular region of subjects undergoing ILM removal. The results demonstrated a limited and delayed recovery of the b-wave amplitude 6 months after surgery. Lim et al. [3] also assessed it by ERG and found that implicit time (time- to-peak of the b-wave) was prolonged, indicating subtle macular dysfunction after ILM peeling. Ramin et al. [6] compared retinal sensitivity and frequency of microscotomas found by SD-OCT combined with SLO microperimetry after idiopathic macular hole closure, in eyes that underwent internal limiting membrane (ILM) peeling and eyes that did not. They found mean retinal sensitivity was lower after ILM peeling and postoperative microscotomas were significantly more frequent. However, one limitation of these studies was inclusion of the macular hole area into analysis when comparing the pre- and post-operation retinal function. It may confound the results. The other limitation of these studies was failure to compare the pre- and post-operative retinal function in a point-to-point pattern due to the inherent limitation of MP-1 and MP-2.

In our study, we assessed the functional changes of the normal retina surrounding the MH after ILM peeling using MP-3. In order to ensure the result gives a strong indication for the effect of ILM peeling on the normal retina, we only choose points in the outer two rings which corresponded to the normal retina surrounding the macular hole, and the area within MH was excluded. There were 28 points in the outer two rings, which occupied 60% of the whole 45 points, but covered more than 75% area of the 8° retina. These points were located from 4° to 8°. The diameter of 8° visual field was 2500um (about 1.6 PD). During the operation, the ILM we peeled off was at least 2 PD, which means the 8° area was completely contained in the ILM peeling area. In the current study, the diameter of the largest MH is 876 μm, which corresponded to approximately central 3.5° in visual field. To further excluding the confounding effect of MH on functional analysis for ILM peeling, the points with a distance from the margin of MH less than 0.5 PD were also excluded.

Patients with severe cataract, which may interfere with the MP-3 measurements (the opacities of all patients’ lens under LOCSIII NO3C2P1 grade), were excluded. Phacoemulsification and IOL implantation were performed in 35 eyes. MRS increased in both groups. The increasing extent of MRS had no difference between the patients with phacoemulsification and those without, suggesting that opacity of lens was not severe in patients with phacoemulsification and this extra procedure did not influence the results.

In the current study, retinal sensitivity in ILM peeling area increased at both 1 and 4 months postoperatively. The reason for this unexpected result in our research might be as following. Firstly, …” this was a short-term study. We only observed the changes in retinal function for 4 months after surgery. The IM peeling procedure itself could be an injury to motivate retinal neural protection and lead to the release of neural protective factions [20, 21]. These factors might improve retinal function in a short-term. If the retinal function was observed for a longer time (such as more than 6 months), the result might be different. Secondly, in the current study, retinal function was evaluated by MP-3. Compared with MP-1 and MP-2, MP-3 has auto tracking and auto aligment, fixation test, wider measurement range, higher resolution non-mydriatic fundus camera and a better system to accomplish the images for pre- and post-treatment comparison. These techniques enable us to do more accurate assessment of macular function. At last, there are often retinal cysts around the margin of macular holes. These cysts can decrease retinal sensitivity. It has been proved that the elimination of retinal cysts followed by MHs healing can increase retinal sensitivity of corresponding area [22]. Although we chose the outer rings to avoid the influence of the function change around the hole as much as possible, it still may cover some areas of the retinal cysts, which may affect the results of normal function measurement.

The post-operative MRS in the selected area increased in 37 patients and deceased in 7 patients. Patients with decreased MRS were significantly older than other patients. We think the reason may be related with retinal recovery ability. Patients with younger age may have a better recovery ability in RS than aged patients. If the sample enlarged, the result might be different.

The pre-operative MRS had no difference between superior and inferior retina or between nasal and temporal retina pre-operation. While the increasing extent of retinal sensitivity in superior retina was significantly higher than that in inferior retina. When performing ILM peeling, the surgeon used to start from superior retinal area. The initiation of ILM peeling may bring more mechanical injury to the superior retina. It may be the reason of this phenomenon. We also found the increasing extent of retinal sensitivity in temporal retina was significantly lower than that in nasal retina. Takayuki et al. [23] had the similar result. They performed vitrectomy and ILM peeling on 39 eyes with MH, and found the retinal sensitivity was significantly lower in the temporal area than in the other areas 3 and 6 months after surgery. The reason for this restricted change to the temporal retina might be as following. Firstly, the removal of the ILM started from the temporal superior retina to the fovea. Secondly, the nerve fiber layer has been reported to be thinnest in temporal quadrant around fovea [24]. Thirdly, the density of ganglion cells at the temporal retina is less than that at the nasal retina within 2 mm from fovea [25].

The limitations of the current study included lack of a control group. A prospective randomized control study is indicated in the future to draw more definitive conclusion.

## Conclusion

ILM peeling in normal retina did not decrease the retinal function in a short-term after surgery, except in some patients with older age. During the surgery, we didn’t use any dye, whose retinal toxicity still needs further study. ILM peeling alone is a safe and useful technique in surgeries for closing macular hole.

## Data Availability

The datasets used and/or analyzed during the current study are available from the corresponding author on reasonable request.

## Abbreviations

ILM: Internal limiting membrane
MH: Macular hole
MP-3: Microperimetry-3
BCVA: Best-corrected visual acuity
MRS: Mean retinal sensitivity
SD-OCT: Spectral domain optical coherence tomography
OCT: Optical coherence tomography
LOCSIII: Lens Opacities Classification System III
IOL: Intra ocular lens
FL: Fixation loss
FP: False-positive
DONFL: Dissociated optic nerve fiber layer
SLO: Scanning laser ophthalmoscopy
FMERGs: Focal macular electroretinograms
